# Estrogen Receptor Subtypes Elicit a Distinct Gene Expression Profile of Endothelial-Derived Factors Implicated in Atherosclerotic Plaque Vulnerability

**DOI:** 10.3390/ijms231810960

**Published:** 2022-09-19

**Authors:** Narjes Nasiri-Ansari, Eliana Spilioti, Ioannis Kyrou, Vassiliki Kalotychou, Antonios Chatzigeorgiou, Despina Sanoudou, Karin Dahlman-Wright, Harpal S. Randeva, Athanasios G. Papavassiliou, Paraskevi Moutsatsou, Eva Kassi

**Affiliations:** 1 Department of Biological Chemistry, Medical School, National and Kapodistrian University of Athens, 11527 Athens, Greece; 2 Laboratory of Toxicological Control of Pesticides, Scientific Directorate of Pesticides’ Control and Phytopharmacy, Benaki Phytopathological Institute, 14561 Athens, Greece; 3 Warwickshire Institute for the Study of Diabetes, Endocrinology and Metabolism (WISDEM), University Hospitals Coventry and Warwickshire NHS Trust, Coventry CV2 2DX, UK; 4 Warwick Medical School, University of Warwick, Coventry CV4 7AL, UK; 5 Laboratory of Dietetics and Quality of Life, Department of Food Science and Human Nutrition, School of Food and Nutritional Sciences, Agricultural University of Athens, 11855 Athens, Greece; 6 Centre for Sport, Exercise and Life Sciences, Research Institute for Health & Wellbeing, Coventry University, Coventry CV1 5FB, UK; 7 Department of Internal Medicine, Laikon General Hospital, Medical School, National and Kapodistrian University of Athens, 11527 Athens, Greece; 8 Department of Physiology, Medical School, National and Kapodistrian University of Athens, 11527 Athens, Greece; 9 Clinical Genomics and Pharmacogenomics Unit, 4th Department of Internal Medicine, Attikon Hospital Medical School, National and Kapodistrian University of Athens, 11527 Athens, Greece; 10 Center for New Biotechnologies and Precision Medicine, Medical School, National and Kapodistrian University of Athens, 11527 Athens, Greece; 11 Biomedical Research Foundation of the Academy of Athens, 11527 Athens, Greece; 12 Department of Biosciences and Nutrition, Novum, Karolinska Institute, SE-14183 Huddinge, Sweden; 13 Endocrine Unit, 1st Department of Propaedeutic Internal Medicine, Laiko General Hospital, National and Kapodistrian University of Athens, 11527 Athens, Greece

**Keywords:** atherosclerosis, plaque vulnerability, estrogen, estrogen receptors, GPR-30, endothelial cells, matrix metalloproteinases MMPs, MCP-1, p21

## Abstract

In the presence of established atherosclerosis, estrogens are potentially harmful. MMP-2 and MMP-9, their inhibitors (TIMP-2 and TIMP-1), RANK, RANKL, OPG, MCP-1, lysyl oxidase (LOX), PDGF-β, and ADAMTS-4 play critical roles in plaque instability/rupture. We aimed to investigate (i) the effect of estradiol on the expression of the abovementioned molecules in endothelial cells, (ii) which type(s) of estrogen receptors mediate these effects, and (iii) the role of p21 in the estrogen-mediated regulation of the aforementioned factors. Human aortic endothelial cells (HAECs) were cultured with estradiol in the presence or absence of TNF-α. The expression of the aforementioned molecules was assessed by qRT-PCR and ELISA. Zymography was also performed. The experiments were repeated in either ERα- or ERβ-transfected HAECs and after silencing p21. HAECs expressed only the GPR-30 estrogen receptor. Estradiol, at low concentrations, decreased MMP-2 activity by 15-fold, increased LOX expression by 2-fold via GPR-30, and reduced MCP-1 expression by 3.5-fold via ERβ. The overexpression of ERα increased MCP-1 mRNA expression by 2.5-fold. In a low-grade inflammation state, lower concentrations of estradiol induced the mRNA expression of MCP-1 (3.4-fold) and MMP-9 (7.5-fold) and increased the activity of MMP-2 (1.7-fold) via GPR-30. Moreover, p21 silencing resulted in equivocal effects on the expression of the abovementioned molecules. Estradiol induced different effects regarding atherogenic plaque instability through different ERs. The balance of the expression of the various ER subtypes may play an important role in the paradoxical characterization of estrogens as both beneficial and harmful.

## 1. Introduction

Atherosclerosis is known to be the major cause of coronary artery disease (CAD), which remains amongst the most prevalent diseases and is the leading cause of death among women in developed countries, such as USA [1]. The prevalence of atherosclerosis was reported as 101.11/per 1000 individuals in 2015 [2] and it was rated as the second leading cause of death following cancer in Canada, with the economic burden of USD 66.6 billion CAD spent between 2005 and 2016 [3]. Atherosclerosis is a systematic inflammatory process which implicates cells of both the immune system and vessel walls. It is considered as the underlying cause of cardiovascular disease (CVD) mortality in both men and women, although, at younger ages, men are at a higher risk of CVD than women of the same age [4,5,6]. Compared to women of reproductive age, the risk of atherosclerosis is significantly increased in women after menopause due to prolonged estrogen deficiency [7,8]. The homeostasis of estrogens is strongly regulated by the balance between its synthesis and deactivation. Decreased circulating estrogen levels, along with estrogen sulfotransferase (SULT1E1), in diabetic postmenopausal women [9] may contribute to an increased risk of atherosclerosis development [9,10]. Interestingly, increased SULT1E1 expression has been found in the atheromatic plaque of both mice and humans, as compared to normal arteries [11]. Notably, SULT1E1 is a key enzyme known to catalyze the sulfation of estrogens, leading to its inactivation [9].

The exogenous administration of estrogen as a hormone-replacement therapy (HRT) is commonly prescribed for postmenopausal women in order to ameliorate the risk of estrogen deficiency-related diseases [12].

The atherogenic process evolves in different stages, starting with endothelium activation/dysfunction and ending with atherosclerotic plaque vulnerability and rupture [13]. Although plaque rupture remains the main plaque complication, other recently identified mechanisms, such as calcified nodules protruding into the artery lumen, have been associated with coronary thrombosis and sudden death [14].

Endothelium is the key vessel wall component involved in the initiation of the atherosclerotic process, while its possible role in the later stages has been widely hypothesized, since the major part of the luminal surface of the artery coated with advanced atherosclerotic plaque is still covered by the intact endothelium, although an area of endothelial denudation can also be detected [15]. It should be noted that the influence of estrogen is highly dependent not only on the cell type but also on its “environment”, with atherogenic plaque representing a special environment containing an extracellular matrix (ECM) under the endothelial layer.

The beneficial actions of estrogen in various tissues and organs, including the cardiovascular system, have been widely recognized. Several observational studies have shown that estrogens provide protection against CVD during the postmenopausal period [7,16]. Concerning the role of phytoestrogens in atherosclerosis progression, the vast majority of clinical studies have shown that phytoestrogen supplementation exerts a rather modest beneficial effect in reducing the CVD risk profile of postmenopausal women, mostly through influencing the blood lipid profile and biomarkers of the endothelial function, while in women with an increased risk of atherosclerosis, a harmful effect on CIMT (carotid intima-media thickness) progression may be observed [17]. Data from animal studies indicated that the treatment of ovariectomized rats with fresh soy oil (phytoestrogen) resulted in the improvement of the atherosclerosis-related blood lipid profile [18], as well as the atherosclerotic plaque’s inflammatory and antioxidant status [19]. However, it should be noted that the repetitive consumption of heated soy oil increased the serum parameters related to atherosclerosis in ovariectomized rats [18].

Moreover, randomized prospective controlled studies failed to confirm the benefits of hormone replacement therapy (HRT) in regard to the primary and secondary prevention of cardiovascular events in postmenopausal women. On the contrary, treatment with estrogen was found to potentially increase the risk of CAD, thus leading to a dramatic decrease in its use [20,21,22,23]. The discrepancy between observational and clinical trials may be related, among other factors, to cardiovascular comorbidities, the age at treatment initiation, and time since menopause—a hypothesis that has been referred as “the timing hypothesis”. Thus, HRT appears to be relatively safe only in younger women who are asymptomatic for CVD and within 10 years away from menopause [23,24,25]. This suggests that, after the onset of atherosclerosis and in the presence of atherosclerotic plaque, estrogen may be potentially harmful. Although the protective effects of estrogens in the early stages of the atheromatosis process (endothelium activation/dysregulation) have been extensively investigated in both in vivo (animal models and clinical studies) and in vitro studies [24,26,27,28,29], data regarding their influences on factors implicated in the later stages of the atherosclerosis process which lead to the plaque vulnerability are limited. It has been hypothesized that the altered expression of estrogen receptors (ER) ERα, Erβ, and GPR-30 on the vascular wall, along with the atherosclerotic lesions following estrogen deprivation, is strongly involved [16,26,30,31,32].

During the stages of plaque rupture and/or erosion, among other factors, monocyte chemoattractant protein-1 (MCP-1) and inflammatory cytokines such as tumor necrosis factor-α (TNF-α) promote atherosclerotic plaque vulnerability through processes such as intimal thickening, ECM degradation, vascular mineralization, and calcification within the atheroma [33]. Recent data indicate the existence of a possible link between circulating MCP-1 levels and the risk of stroke and coronary artery disease [34,35,36]. Moreover, MCP-1 plaque levels have been associated with histopathological hallmarks of plaque vulnerability [36]. The metalloproteinases MMP-2 and MMP-9, as well as their inhibitors, TIMP-1 and TIMP-2, are both expressed in the endothelial cells [37,38].

Recent data point toward a close association of the expression and activity of matrix metalloproteinases (MMPs) with plaque stability and the consequent incident of cardiovascular complications, since they regulate the collagen degradation of the ECM [33,37,38,39,40]. The ratio between the expression and activity of MMPs and their inhibitors, known as the tissue inhibitors of metalloprotease (TIMPs), has been used as a critical indicator of CVD pathogenesis and atherosclerotic plaque instability in both human and animal studies [38,41]. Independently of the roles of TIMP on MMP inhibition, TIMP-2 mediates the G1 cell cycle arrest [42,43] by binding to human endothelial cells through α3/β1, leading to decreased angiogenesis and cell proliferation [14,43]. Notably, the inhibition of cycle progression during the G1 phase of the cell cycle by the inactivation of cyclin-cyclin-dependent kinase (CDK) complexes contributes to reduced atherosclerotic plaque formation and neointimal thickening [44].

ECM proteins are also regulated by metalloproteinases with thrombospondin motifs, such as ADAMTS family members. ADMTS-4 has recently emerged as an important player in the atherosclerosis process [45]. Interestingly, in ADAMTS-4 knockout (KO) mice, a decrease in high fat diet-induced atherosclerosis and increased plaque stability were observed [46]. A positive correlation of serum levels of ADAMTS-4 with an increased risk of developing CAD has been proved in regard to various patient groups with different underlying diseases [45].

Platelet-derived growth factor-β (PDGF-β) is also expressed by endothelial cells and regulates the atherosclerosis progression and the plaque stability. Notably, AG1296, a potent tyrosine kinase inhibitor which can block the PDGF-PDGFR signaling pathway, was found to enhance plaque stability via, among other mechanisms, the reduction in the expression of MMP-2 and MMP-9 [47].

Lysyl oxidase (LOX) is also involved in the process of plaque vulnerability. Low LOX activity can lead to defective collagen cross-linking, which in turn can weaken the fibrous cap and favor the presence of soluble forms of collagen which are highly susceptible to metalloproteinase degradation. Indeed, higher levels of LOX have been detected in more stable plaques [48,49].

Moreover, plaque calcification—a lesion associated with coronary thrombosis, even in the absence of eroded or ruptured plaque—is directly linked to the imbalance between the receptor activator of the nuclear factor NFκB ligand (RANKL) and osteoprotegerin (OPG), which are also expressed in endothelial cells [50,51,52].

OPG, a secreted member of the TNF receptor family, is a decoy receptor for RANKL and inhibits the initiation of RANK signaling. Interestingly, OPG knockout mice displayed pronounced arterial calcification [53]. It has also been revealed that OPG exerts anti-apoptotic effects on endothelial cells, acting as an autocrine survival factor [54,55]. Since endothelial apoptosis precedes vascular calcification [56], OPG may exert its protective effect in vascular calcification through its anti-apoptotic action [57].

On the other hand, the overexpression of RANKL, which is also expressed in the endothelial cells, was found to elevate MMP-9 activation [52,58,59]. The aforementioned effect of RANKL is neutralized by OPG through the inhibition of the RANK/RANKL interaction [60]. The reduced OPG/RANKL ratio can indirectly increase metalloproteinase activity, leading to atherosclerotic plaque erosion and rupture [61]. Clinical studies have confirmed a strong association between OPG and soluble RANKL serum levels in CAD [62,63]. Moreover, OPG-induced LOX upregulation has been linked to the formation of stable fibrous caps in APOE knockout mice [64,65].

Recent studies have suggested the implication of the onco-suppressor p53 and its transcriptional target p21 in the atherogenesis process, as well as in plaque calcification [66,67]. The inactivation of p21 appears to exert atheroprotective effects by inhibiting lesion growth and the maintenance of atherosclerotic plaque stability [68]. Studies on tissues and organs affected by estrogen functions (both normal and neoplastic) have shown that estrogen regulates the expression of *p53* and *p21*; however, data regarding their estrogen-regulated expression in the vascular wall components that participate in the atherogenesis process are lacking [69,70,71].

While the protective effects of estrogens, with the crucial role of ERα, in the early stages of the atheromatosis process (endothelium activation/dysregulation) have been extensively investigated using both in vivo and in vitro approaches [8,72,73,74], data on estrogen’s influences on factors implicated in the later stages of the atheromatosis process which lead to plaque vulnerability are limited. It has been hypothesized that the altered expression of estrogen receptors on the vascular wall, following estrogen deprivation, as well as the altered expression of estrogen receptors (ERα, Erβ, and GPR-30) in the atherosclerotic lesion, are implicated [26,31,32,75]. A previous study of the other types of cells besides those implicated in the atherosclerosis process (fibrochondrocytes) demonstrated that E_2_ mediates MMP-9 overexpression through the activation of the ERα/ERK and NF-κB/ELK-1 signaling pathways [76]. Specifically, in the presence of E_2_, ERα induces ERK phosphorylation, leading to the further activation of its downstream targets, such as NF-κB or ELK-1, and finally triggers MMP-9 overexpression. Interestingly, this effect was diminished in cells carrying mutations in the NF-κB or ELK-1 binding sites [76]. However, other studies have demonstrated that the effects of E_2_ on NF-κB activity and MMP expression are cell-specific and, at least in part, depend on E_2_ concentrations [77,78].

To this end, in this study, we aimed to investigate the effects of various concentrations of estradiol, either alone or after mimicking a low-grade inflammation state that occurs post-menopause in established atherosclerosis, on the expression of molecules involved in atherosclerosis plaque vulnerability (MCP-1, PDGF-β, ADAMTS-4, MMP-2, MMP-9, TIMP-1, TIMP-2, and OPG/RANK/RANKL expression) and MMP activity, using human aortic endothelial cells (HAECs), which offer the best in vitro model system for studying CVD. Moreover, we aimed to clarify whether these effects are mediated by the ERα, Erβ, or GPR-30 receptors. Finally, we delineated the role of p21 in the estrogen-mediated regulation of the expression of the aforementioned genes in the endothelial cells.

## 2. Result

### 2.1. The Incubation of HAEC Cells with Estradiol and TNF-α Had No Effect on Cell Viability

**Figure 1:**
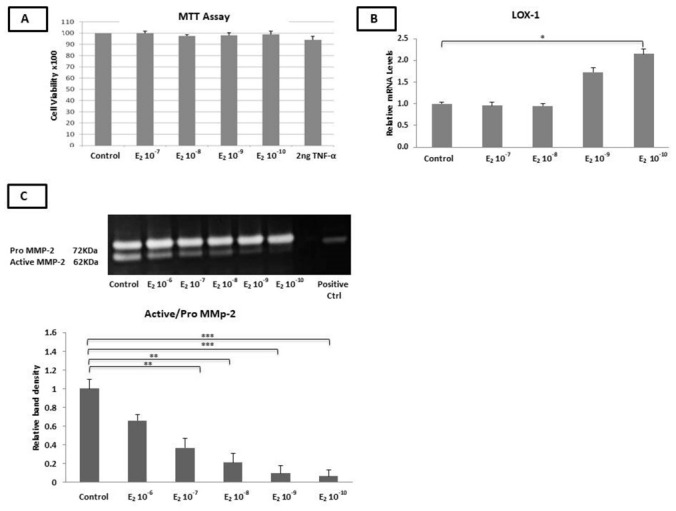
Effect of treatment with 10^−10^−10^−7^ M E_2_ on cell survival, atherosclerotic gene expression, and MMP-2 gelatinase activity. (**A**) MTT assay. Incubation of HAECs with 10^−10^–10^−7^ M E_2_ or 2 ng/mL TNF-α for 24 h had no significant effect on cell viability. (**B**) LOX-1 mRNA levels were significantly increased in the presence of 10^−10^ M E_2_ over 24 h. (**C**) The ratio of active MMP-2/pro-MMP-2 was significantly reduced after 24 h of incubation with various concentrations of E_2_. The graphical data are represented as mean ± SD of at least three independent experiments (*** *p* < 0.001, ** *p* < 0.01, * *p* < 0.05).

HAECs were treated with either 17β-estradiol (E_2_) (10^−10^–10^−7^ M) or TNF-α (2 ng/mL) for 24 h (24 h). A cell proliferation assay revealed that neither E_2_ nor TNF-α had a significant impact on HAEC viability (Figure 1A).

### 2.2. Estradiol Did Not Alter the mRNA Levels of RANK, OPG, and MCP-1 and the TIMP-1, TIMP-2, and MCP-1 Protein Levels

HAEC cells were incubated with E_2_ (10^−10^−10^−7^ M) for 6 h and 24 h.

The incubation of the cells with E_2_ alone for 6 h had no significant effect on the expression of LOX, RANK, RANKL, OPG, MMP-2, MMP-9, TIMP-1, TIMP-2, PDGF-β, ADAMTS-4, and MCP-1 as compared to the untreated cells.

The incubation of the cells with E_2_ for 24 h significantly increased the mRNA expression of LOX (10^−10^ M, *p* < 0.05) (Figure 1B), while the mRNA expression of the RANKL, MMP-2, MMP-9, TIMP-1, TIMP-2, OPG, PDGF-β, RANK, ADAMTS-4, and MCP-1 genes remained unchanged.

The protein levels of secreted TIMP-1, TIMP-2, and MCP-1 were not significantly altered after the incubation of the HAECs with various concentrations of E_2_ for 6 h and 24 h, while the expression of secreted OPG was not detected under our experimental conditions, as assessed by ELISA.

### 2.3. Estradiol Reduced the MMP-2 Gelatinase Activity

The incubation of the HAECs with E_2_ (10^−10^−10^−6^ M) for 24 h resulted in a significant reduction in the active form of MMP-2 in a dose-dependent manner, with stronger effects at the lower concentrations of E_2_ (10^−7^–10^−8^ M, *p* < 0.01 and 10^−9^–10^−10^ M, *p* < 0.001), compared to the untreated cells (Figure 1C) (*p* < 0.05).

### 2.4. Estradiol Altered the mRNA Expression of LOX-1, TIMP-1, MMP-9, and MCP-1 and MCP-1 Protein Levels under Inflammatory Conditions

**Figure 2:**
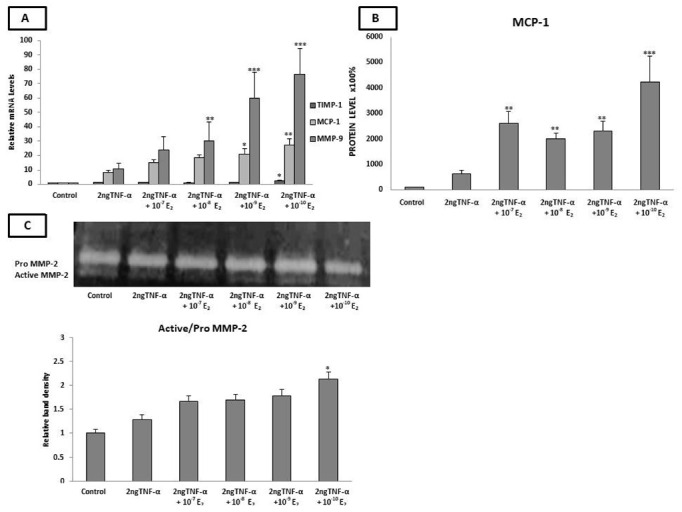
Effects of the pre-incubation of HAECs with 2 ng TNF-α followed by co-incubation of the cells with E_2_ on the expression of genes and protein involved in plaque stability, as well as on MMP-2 gelatinase activity. (**A**) Pre-incubation of HAECs with 2 ng/mL TNF-α for 24 h followed by co-the incubation of cells with 10^−10^–10^−7^ M E_2_ for a further 24 h significantly increased the expression of TIMP-1, MCP-1, and MMP-9, as compared to cells incubated with TNF-α alone. (**B**) Pre-incubation of HAECs with 2 ng/mL TNF-α for 24 h followed by the co-incubation of cells with 10^−10^–10^−7^ M E_2_ for a further 24 h significantly increased the MCP-1 protein levels as compared to cells incubated with TNF-α alone. (**C**). The ratio of active MMP-2/pro-MMP-2 was significantly increased after 24 h incubation with 2 ng/mL TNF-α followed by the co-incubation of cells with 10^−10^ E_2_. The graphical data are represented as mean ± SD of at least three independent experiments (*** *p* < 0.001, ** *p* < 0.01, * *p* < 0.05).

In order to mimic the low-grade inflammation state that exists in postmenopausal women [79], we pre-incubated the endothelial cells with TNF-α for 24 h followed by co-incubation with E_2_ for another 24 h. The RT-PCR analysis revealed a significant increase in the mRNA levels of MMP-9 (10^−8^ M, *p* < 0.01 and 10^−9^–10^−10^ M, *p* < 0.001), TIMP-1 (10^−10^ M, *p* < 0.05), and both the mRNA (10^−9^ and 10^−10^ M, *p* < 0.05 and *p* < 0.01 respectively) and protein levels (10^−7^–10^−10^ M, *p* < 0.01) of MCP-1 as compared to cells incubated with TNF-α alone (Figure 2A,B).

No significant effect was observed on the RANK, RANKL, MMP-2, ADAMTS-4, PDGF-β, and LOX mRNA levels, as well as on the TIMP-1,TIMP-2, and OPG protein levels.

### 2.5. Estradiol Induced MMP-2 Activity under Low-Grade Inflammatory Conditions

The matrix metalloproteinase activity was evaluated in cells pre-incubated with 2 ng TNF-α for 24 h and co-incubated with E_2_ (10^−10^−10^−7^ M) for a further 24 h. As shown in Figure 2C, the incubation of cells with TNF-α alone induced MMP-2 activity as compared to untreated cells. A significant increase in MMP-2 activity was detected after the co-incubation of the cells with E_2_ (10^−10^ M) as compared to cells incubated with TNF-α alone (*p* < 0.05). Notably, MMP-9 enzymatic activity was not detected in the presence of TNF-α using gelatin zymography.

### 2.6. HAECs Express the GPR30 Estrogen Receptor

**Figure 3:**
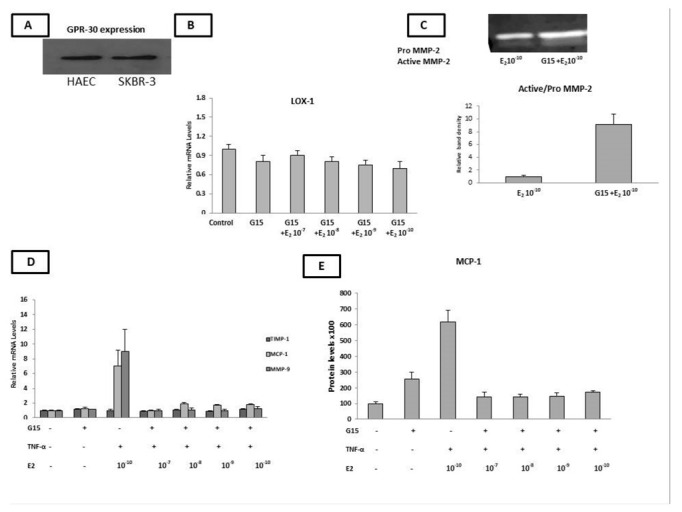
HAECs were co-incubated with G15 (GPR-30 antagonist) and various concentrations of E_2_ in the presence/absence of low-grade inflammation (TNF-α). (**A**) GPR-30 protein levels were detected by western blotting in HAECs. (**B**) Co-incubation of HAECs with G15 and E_2_ reversed the E_2_-induced increase in LOX-1 mRNA levels (10^−10^ M of E_2_). (**C**) Co-incubation of HAECs with G15 reversed the E_2_-reduced active/proMMP-2 ratio (10^−10^ M of E_2_). (**D**) Pre-incubation of cells with TNF-α followed by the co-incubation of cells with G15 and various concentrations of E_2_ reversed the (TNF-α + E_2_)-induced increase in TIMP-1, MCP-1, and MMP-9 mRNA levels. (**E**) Pre-incubation of cells with TNF-α followed by the co-incubation of cells with G15 and various concentrations of E_2_ reversed the (TNF-α + E_2_)-induced increase in MCP-1 protein levels. The graphical data are represented as mean ± SD of at least three independent experiments.

Next, we aimed to investigate whether the observed changes in the expression of molecules implicated in the advanced stages of the atherosclerosis process upon treatment with E_2_ were mediated through Erα-, Erβ-, or GPR-30-dependent pathways. Thus, the basal mRNA levels of ERα, Erβ, and GPR-30 were evaluated by qPCR. Interestingly, the HAECs expressed high levels of GPR-30 (Figure 3A), while the mRNA levels of ERα were faintly detected. ERβ mRNA levels were undetectable (Ct = 37) in the HAECs (Supplementary Figure S1). Furthermore, the ERα and ERβ proteins were not detected in the HAEC cells by western blot analysis (Supplementary Figure S1), while the GPR-30 protein was highly expressed (Figure 3A).

Notably, the Flag-ERβ MCF-7-tet-off breast cancer cell line was used as a positive control for the detection of both the ERα and ERβ mRNA levels, while SKBR-3 cells were used as a positive control for the detection of GPR-30.

### 2.7. G15 (GPR-30 Antagonist) Countered the Estradiol-Induced Expression of LOX, MCP-1,TIMP-1, MMP-9, and MCP-1 as Well as the Decreased MMP-2 Gelatinase Activity

Given that GPR-30 was the only ER expressed in our HAECs, we co-incubated cells with an E_2_- and GPR-30-specific antagonist, G15 (10^−6^ M), in order to confirm that E_2_ exerted its effects via GPR-30.

We found that G15 countered the effect of E_2_ in inducing LOX mRNA expression (Figure 3B) and reversed the E_2_-dependent reduction in the MMP-2 enzymatic activity in the HAECs (Figure 3C).

We then investigated the effect of G15 (10^−6^ M) on the observed changes in the expression of MCP-1, TIMP-1, and MMP-9 when the cells were pre-incubated with TNF-α (2 ng for 24 h) and co-incubated with various concentrations of E_2_ (for a further 24 h). In the presence of G15, the mRNA levels of TIMP-1 (10^−10^ M of E_2_) and MMP-9 (10^−8^–10^−10^ M of E_2_), as well as the mRNA and protein levels of MCP-1 (10^−10^–10^−7^ M of E_2_), which were increased after the co-incubation of the HAECs with TNF-α and E_2_, reversed to the basal levels (Figure 3D,E).

These results indicate the E_2_ (alone or in the presence of an inflammatory stimulus (TNF-α)) regulates the expression of the LOX-1 mRNA levels, MMP-2 gelatinase activity, and mRNA levels of LOX-1, MCP-1, MMP-9, TIMP-1, as well as the MCP-1 protein levels and MMP-2 gelatinase activity, respectively, through binding to GPR-30.

### 2.8. Estradiol Increased the Expression of MCP-1 and TIMP-1, as Well as MMP-2 Enzymatic Activity, through ERα

To determine the roles of the other two estrogen receptors (ERα and ERβ) in the regulation of molecules involved in the formation and stability of atherosclerotic plaque, we transfected HAECs with plasmids expressing either ERα or ERβ and their corresponding vectors, since both ERs were not expressed in the HAECs. Twenty-four hours after transfection with either plasmid, the transfection efficiency was evaluated by qPCR analysis (Supplementary Figure S1).

**Figure 4:**
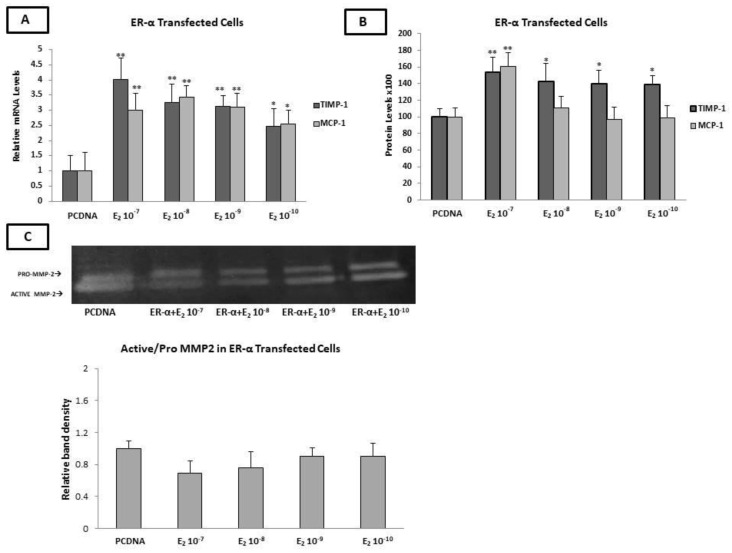
Effects of E_2_ on the expression of factors involved in plaque stability in Erα-transfected HAECs. (**A**) Incubation of ER-transfected HAECs with E_2_ induced an increase in the mRNA levels of TIMP-1 and MCP-1. (**B**) Incubation of ERα-transfected HAECs with E_2_ induced an increase in TIMP-1 (at all concentrations of E_2_) and MCP-1 (only at the concentration of 10^−7^ M of E_2_) protein levels. (**C**) Incubation of ERα-transfected HAECs with E_2_ had no effect on the MMP-2 gelatinase activity. The graphical data are represented as mean ± SD of at least three independent experiments (** *p* < 0.01, * *p* < 0.05).

At twenty-four hours post-transfection, the ERα-transfected HAECs were incubated with E_2_ (10^−10^–10^−7^ M) for a further 24 h. The qPCR analysis revealed that E_2_ significantly increased the expression of TIMP-1 and MCP-1 (10^−7^–10^−9^ M of E_2_ *p* < 0.01 and 10^−10^ M of E_2_, *p* < 0.05 for both genes) in ERα-transfected HAECs (Figure 4A). These findings were confirmed at the protein level for MCP-1 only at the highest concentration of E_2_ (10^−7^ M, *p* < 0.01) and for TIMP-1 at all concentration ranges of E_2_ (10^−7^ M *p* < 0.01 and 10^−8^ M–10^−10^ M, *p* < 0.05) after 24 h of incubation (Figure 4B). No significant changes in the protein expression of TIMP-2 was detected after the incubation of the Erα-transfected HAECs with different concentrations of E_2_.

Moreover, in ERα-transfected HAECs cells, the MMP-2 gelatinase activity was not altered upon incubation with various concentrations of E_2_ (Figure 4C).

### 2.9. Estradiol Reduced the PDGF-β mRNA Levels and MCP-1 Protein Levels through ERβ

As has already been mentioned, the applied HAECs did not express either of the two nuclear estrogen receptors. Therefore, we transfected cells with ERβ-GFP-tagged plasmid, and the transfection efficiency was determined by measuring the mRNA and protein levels of ERβ by both qPCR and western blot, respectively.

**Figure 5:**
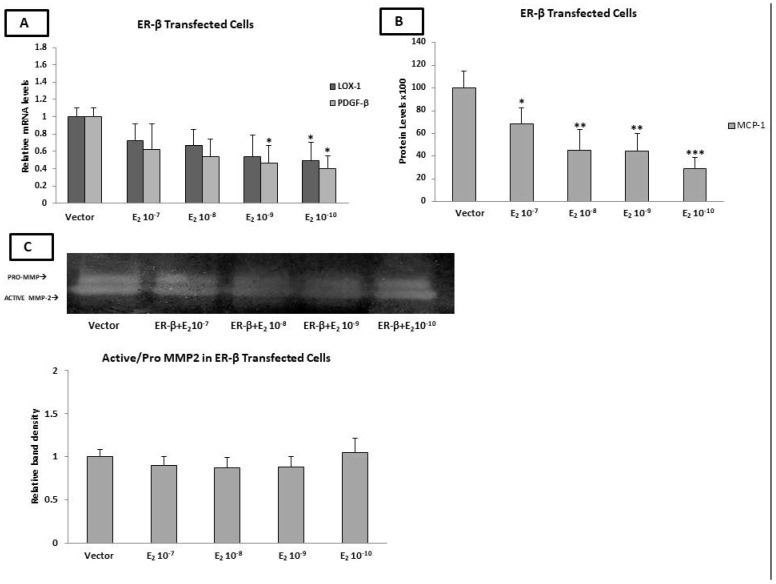
Effect of E_2_ on the expression of factors involved in plaque stability in ERβ-transfected HAECs. (**A**) Incubation of ERβ-transfected HAECs with E_2_ reduced the mRNA levels of LOX-1 (only at 10^−10^ M of E_2_) and PDGF-β (10^−9^ and 10^−10^ M of E_2_). (**B**) Incubation of ERβ-transfected HAECs with E_2_ (10^−7^–10^−10^ M) reduced MCP-1 protein levels. (**C**) Incubation of ERβ-transfected HAECs with E_2_ had no effect on MMP-2 gelatinase activity. The graphical data are represented as mean ± SD of at least three independent experiments (*** *p* < 0.001, ** *p* < 0.01, * *p* < 0.05).

ERβ-transfected HAECs were incubated with E_2_ (10^−10^−10^−7^ M) for 24 h. As shown in Figure 5A, E_2_ significantly reduced the LOX and PDGF-β mRNA levels at concentrations of 10^−10^ M and 10^−10^−10^−9^ M, respectively (*p* < 0.05), while no significant changes were observed in the MCP-1 mRNA levels.

The incubation of ERβ-transfected HAECs with E_2_ (10^−10^−10^−7^ M) resulted in no significant changes in the mRNA levels of MMP-2, MMP-9, TIMP-1, TIMP-2, MCP-1, and ADAMST-4. The mRNA expression of OPG was undetectable after transfection with ERβ.

Interestingly, the ELISA demonstrated that the incubation of ERβ-transected HAECs with various concentrations (10^−10^−10^−7^ M) of E_2_ resulted in a significant decrease in the MCP-1 protein levels (10^−7^ M of E_2_ *p* < 0.05 and 10^−8^–10^−9^ M of E_2_ *p* < 0.01), with the more pronounced effect being exhibited at the lowest concentration of E_2_ (10^−10^ M of E_2_, *p* < 0.001). No significant changes in the protein expression of TIMP-1 and TIMP-2 were found after the incubation of the ERβ-transfected HAECs with different concentrations of E_2_. Notably, OPG protein was not detected by ELISA in the cell supernatant (Figure 5B).

The incubation of ERβ-transfected cells with E_2_ (10^−10^–10^−7^ M) did not change the MMP-2 enzymatic activity, as demonstrated by gelatin zymography. It is noteworthy that, under this condition, the MMP-9 pro-enzyme and active enzyme were not detected (Figure 5C).

### 2.10. The p21 Silencing Reduced the Estradiol-Mediated Upregulation of MCP-1 and LOX-1, while It Increased the PDGF-β mRNA Levels

Previous studies have shown that the activation of GPR-30 leads to the upregulation of p21 [80,81]. The overexpression of p21 significantly reduced the expression of vascular cell adhesion molecule 1 (VCAM-1), inhibiting monocyte adhesion. These data suggest that p21 plays an important role in the process of atherogenesis [82]. However, data regarding the role of p21 in atherosclerosis plaque stability are lacking.

**Figure 6:**
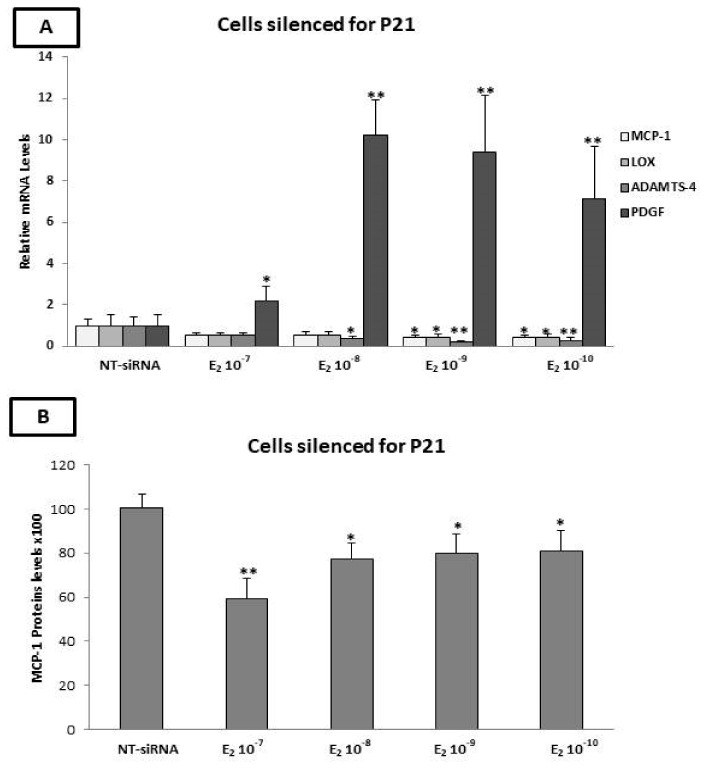
Effect of E_2_ on the expression of factors involved in plaque stability in p21-silenced HAECs. (**A**) Incubation of p21-silenced cells with E_2_ resulted in the induction of PDGF-β (10^−10^−10^−7^ M of E_2_) and reduction in MCP-1 (10^−10^−10^−9^ M of E_2_), LOX-1 (10^−10^−10^−8^ M of E_2_) and ADAMTS-4 (10^−10^ M of E_2_) mRNA levels. (**B**) Incubation of p21-silenced cells with E_2_ resulted in reduced MCP-1 protein levels (10^−10^−10^−7^ M of E_2_). The graphical data are represented as mean ± SD of at least three independent experiments (** *p* < 0.01, * *p* < 0.05).

To this end, the expression of p21 was silenced in the HAECs using p21-siRNA. After p21 silencing, the cells were incubated with E_2_ for 24 h. As shown in Figure 6A, significant reductions in the mRNA expression of MCP-1 (10^−9^ and 10^−10^ M of E_2_, *p* < 0.05), LOX (10^−9^ and 10^−10^ M of E_2_, *p* < 0.05), and ADAMST-4 (10^−8^ M of E_2_, *p* < 0.05 and 10^−10^−10^−9^ M of E_2_, *p* < 0.01) were observed. On the contrary, the expression of PDGF-β was significantly increased after the incubation of the cells with E_2_ (10^−7^ M, *p* < 0.05 and 10^−8^–10^−10^ M, *p* < 0.01). No significant changes in the gene expression of MMP-2, MMP-9, TIMP-1, TIMP-2, and RANK were detected. The expression of OPG was undetectable by qPCR after p21 silencing.

### 2.11. Evaluation of MCP-1, TIMP-1, TIMP-2, and OPG Protein Levels by ELISA after p21 Silencing

The effects of p21 silencing on the protein levels of TIMP-1, TIMP-2, MCP-1, and OPG were evaluated by ELISA. The expression of MCP-1 was significantly reduced after the incubation of the cells with E_2_ at concentrations of 10^−7^ M (*p* < 0.01) and 10^−8^–10^−10^ M (*p* < 0.05) for 24 h, while no significant changes in the expression of TIMP-1 and TIMP-2 were detected (Figure 6B). Furthermore, the OPG protein remained undetectable in the cell supernatant, as revealed by ELISA.

## 3. Discussion

The timing hypothesis was first proposed by Thomas Clarkson in 1998 and later confirmed by the Early Versus Late Intervention Trial (ELITE) and Peking studies [83]. According to these studies, the administration of exogenous estrogen to women who have had a menopausal status for approximately ten years can exert adverse effects leading to CVD complications, indicating that, when atherosclerosis is already established, HRT does not provide cardiovascular protection [83]. In line with this hypothesis, both prospective randomized Women’s Health Initiative (WHI) and ELITE studies demonstrated that HRT must be started within 5 to a maximum of 10 years of menopause in order to exert cardio-protective effects without adverse effects [83,84]. However, there are no data regarding the molecular mechanism of estrogen-induced adverse effects on established atherosclerosis. Our in vitro study was designed to investigate the “timing hypothesis” of estrogen replacement therapy at the molecular level and shed light on the specific roles of the different estrogen receptors in the advanced stages of atherosclerosis.

According to our findings, the incubation of HAECs with E_2_ alone, at the lower concentration, increased the expression of LOX, while it exerted no significant effects on the expression of other molecules implicated in the advanced stages of atherosclerosis and plaque vulnerability, such as MMP-2, MMP-9, PDGF-β, TIMP-1, and MCP-1. A study by Sun et al. [85] showed that the incubation of HUVECs with a phytoestrogen, namely pseudoprotodioscin, resulted in a reduction in MCP-1 mRNA expression, an effect mediated by ERα. This inconsistency could be due to differences in the estrogen receptor profiles of our HAECs, which expressed only GPR-30.

We also demonstrated that E_2_ decreased the MMP-2 activity. Interestingly, since our cells express only GPR-30, this favorable effect is exerted via this membrane receptor. Indeed, co-incubation with the antagonist G15 reversed the decrease in the MMP-2 activity.

In order to mimic the low-grade inflammation state that exists in postmenopausal women with atherosclerosis, we pre-incubated the endothelial cells with TNF-α at a low concentration [86,87] and then co-incubated them with E_2_ at various concentrations, including the low concentrations found in the serum of women receiving estrogen replacement therapy.

Interestingly, under low-grade inflammation, E_2_ at low concentrations (10^−10^ M) increased the mRNA and protein levels of MCP-1, which is a key player in atherosclerotic plaque formation and destabilization.

MCP-1 is one of the most widely studied chemokines involved in the atherosclerosis process and has been postulated to be a direct mediator of plaque instability [88]. Interestingly, the local gene silencing of MCP-1 expression turned a vulnerable plaque into a more stable plaque phenotype in ApoE(−/−) mice [89]. The elevated expression of MCP-1 has also been found in the unstable plaques of CVD symptomatic patients [90].

Notably, the co-incubation with the GPR-30 antagonist G15 totally reversed the stimulatory effect of E_2_ on MCP-1 mRNA expression, implying that GPR-30 exerts unfavorable effects of E_2_ on the molecules implicated in the vulnerability of atherosclerotic plaque under low-grade inflammation conditions.

A previous study investigating the effect of E_2_ on MCP-1 expression in breast cancer cell lines reported that E_2_ induced MCP-1 expression when the cells were pre-treated with TNF-α. Notably, these breast cancer cells expressed both GPR-30 and ERα [91]. On the other hand, in rat aortic smooth muscle cells (RASMCs), E_2_ inhibited the stimulatory effect of TNF-α on MCP-1 expression via an ERβ-dependent mechanism [92,93]. Accordingly, herein, we found that ERβ mediates the MCP-1-lowering effects of E_2_, albeit in the absence of inflammatory stimuli.

Our data showed that, in a low-grade inflammation state, E_2_ also reduced LOX-1 expression, an effect mediated via GPR-30. Our current knowledge regarding the role of LOX in the formation, progression, and vulnerability of atherosclerosis plaque is limited and is mostly derived from in vitro studies. Interestingly, a study by Jover et al. revealed that the expression of LOX is linked to plaque stability. As such, low LOX activity could lead to defective collagen cross-linking, resulting in a weaker fibrous cap and plaque instability [48,49]. Previous studies on another type of cells (Ishikawa cells) demonstrated that, in the absence of TNF-α, E_2_ induced LOX-1 expression, while in rat cardiac fibroblasts, it exerted the opposite effect, mediated mainly through ERβ [94,95].

Increased MMP activity is a known indicator of atherosclerotic plaque instability [38,96,97]. Elevated MMP-2 activity has been determined to be an independent mortality marker in patients with acute coronary syndrome [98]. Studies in ApoE-/- mice showed that MMP-2 activity also contributes to the calcification of advanced atherosclerotic lesions [78,79]. Herein, we showed that E_2_ can reduce the activation of MMP-2 in a dose-dependent manner. More interestingly, low concentrations of E_2_ showed a more potent effect on the attenuation of MMP-2 activity. A previous study conducted on smooth muscle cells (ASMC) isolated from B6 mice showed that E_2_ reduced MMP-2 activity, indicating its favorable effects in the absence of inflammation [99]. Wingrove et al. demonstrated that the incubation of human coronary artery vascular smooth muscle (CAVSMC) cells with E_2_ for 72 h resulted in a dose-dependent increase in the expression of pro-MMP-2, an effect that was reversed by the estrogen receptor antagonist tamoxifen [100]. In line with these findings, we found that the expression of pro-MMP-2 was also induced; however, the conversion of pro-MMP2 to active MMP2 was decreased upon treatment with E_2_. Significantly, we found that, in a state resembling low-grade inflammation (such as established atherosclerosis), E_2_ further increased the TNF-α-induced MMP-2 activation in HAECs, with the greatest effect at the lowest concentration.

The expression of MMP-9 was faintly detected in the presence of E_2_ alone, while it was significantly elevated upon treatment of the HAECs with TNF-α alone, as expected [101,102]. Interestingly, we demonstrated that E_2_ further promoted the TNF-α-induced MMP-9 expression in the HAECs. Zanger et al. demonstrated a significant increase in the MMP-9 plasma levels of 10 postmenopausal women with a history of established CAD when receiving oral HRT, which is of clinical relevance to our observation [103]. However, not all clinical studies point towards a harmful effect of oral hormone replacement therapy on the indices of plaque vulnerability [103,104]. This divergency could be attributed to differences in age, the duration of the post-menopausal period, and the existence (or not) of established CVD among the studies’ participants.

It is known that the imbalance between MMPs and TIMPs is implicated in atherosclerotic plaque instability [105]. Reduced TIMP expression has been correlated with unstable plaques and acute coronary syndrome in humans [106]. Our results showed that E_2_ increased the TNFα-induced expression of TIMP-1. However, this elevation did not exceed the MMP-9 overexpression, suggesting that, in the presence of inflammatory stimuli, E_2_ increased the MMP-9/TIMP-1 expression ratio. In accordance with our in vitro model, the MMP-9/TIMP1 ratio was also significantly higher in women receiving estrogen replacement therapy compared to women not taking HRT [107].

All the abovementioned effects were mediated via GPR-30, since the HAECs used for our experiments expressed only this estrogen receptor. Previous studies demonstrated that estrogen can regulate MMP and TIMP expression by binding to either the classical estrogen receptors ERα and ERβ or its membrane-bound estrogen receptor GPER-30 [108]. However, GPR-30 seems to play a prominent role in the regulation of MMPs in the cardiovascular system [109,110]. As such, apigenin, a compound with strong estrogen-like effects, inhibited the MMP-9 expression in endothelial cells through GPR-30 [111], while the incubation of cardiac fibroblasts isolated from Sprague−Dawley rats with G1, an GPR-30 agonist, significantly increased the MMP-2 and reduced TIMP-1 expression [108].

Notably, previous studies demonstrated that estrogen can regulate MMP and TIMP expression by binding to either the classical estrogen receptors ERα and ERβ or its membrane-bound estrogen receptor GPR-30 [108]. To elucidate the specific role of the classical estrogen receptors in the regulation of the abovementioned factors implicated in the later stages of the atherosclerosis process, we transfected HAECs with either ERα or ERβ. Herein, we observed that the transfection of HAECs with either ERα or ERβ did not affect the MMP-2 and MMP-9 expression and gelatinase activity. Although the TIMP-1 and TIMP-2 mRNA levels were elevated significantly after the incubation of Erα-transfected cells with E_2_ at lower concentrations, the protein levels of TMP-1 and TIMP-2 were not increased significantly, suggesting possible post-transcriptional modifications.

Moreover, the incubation of ERα-transfected HAECs with E_2_ resulted in a marginal increase in the LOX-1 and MCP-1 expression. Studies on other estrogen-dependent tissues have shown that E_2_ induces LOX mRNA expression in the uteri of WT mice, as compared to ERαKO mice, indicating the important role of ERα in estrogen-mediated LOX overexpression [112,113]. Moreover, ERα inhibition was found to reduce the MCP-1 expression in mesangial cells [114].

Our data also revealed that the incubation of Erβ-transfected cells with E_2_ results in a reduced PDGF-β and LOX expression. There is evidence to suggest that ERβ reduces the expression and secretion of the pro-agiogenic factors PDGF-β in T47D breast cancer cells [115].

In line with our findings, Iorga et al. showed that DPN treatment, a selective Erβ agonist, in mice with advanced heart failure (HF) significantly reduces the LOX-1 mRNA expression in the ventricles as compared to untreated HF mice [94]. Moreover, the transfection of cells with ERβ resulted in a reduced MCP-1 protein expression, while it had no effect on the TIMP-1 and TIMP-2 protein levels. As shown by Kanda et al., E_2_-bound ERβ restrains the expression of MCP-1 by inhibiting the Sp1 and AP-1 transcriptional activities in keratinocytes [116]. To our knowledge, the present study provides the first experimental evidence to support the regulatory roles of ERβ in PDGF-β and MCP-1 expression in the endothelial cells.

Divergent data exist regarding the role of p21 in the atherosclerosis process and plaque stability, which has been assumed as both proatherogenic and antiatherogenic according to animal studies. However, the majority of the literature points toward a favorable effect; thus, therapies that target p21WAF1 for inactivation, in the appropriate situation, may offer protection against atherosclerosis [66,68,117,118].

Given the equivocal role of p21 in the atherosclerosis process and the fact that E_2_ can exert effects by regulating p21 expression [71], we also aimed to explore the effect of p21 silencing on the estrogen-induced changes in the expression of molecules involved in the later stages of atherosclerosis [68].

Our results illustrate that the incubation of HAECs with E_2_ after p21 silencing results in reductions in the MCP-1, ADAMST-4 and LOX mRNA levels. It is noteworthy that, when p21 is expressed, a low concentration of E_2_ exerts the opposite effects, inducing the expression of LOX-1 in HAECs. As addressed in the literature, p21 positively affects the expression of MCP-1 in endothelial cells [119]. Additionally, it has been shown that the activation of GPR-30 (via the G1 agonist) increases the expression of p21 in breast cancer cell lines [81]. Given that GPR-30 is the only estrogen receptor expressed in our cells, it appears that the increase in the LOX expression by E_2_ is mediated by GPR-30 through p21, thus providing a possible explanation for the effect of p21 silencing, which resulted in reduced LOX expression. It should be noted that, in the literature thus far, there are no data regarding the regulation of LOX by p21 in human endothelial cells. Another notable finding of our study is that p21 silencing resulted in a significant increase in PDGF-β expression in HAECs upon treatment with E_2_, which may contribute to plaque instability [120]. Based on these findings, it could be suggested that the expression of PDGF-β, LOX -1, and MCP-1 is regulated by estrogen through more than one mechanism, including a p21-dependent mechanism. Moreover, p21 silencing may change the balance of these multiple (counter-)regulatory mechanisms, thus resulting in either an atheroprotective effect by decreasing the expression of LOX-1, MCP-1, and ADAMTS-4 or an increased risk of atheromatic plaque rupture through the elevation of PDGF-β expression, depending on the actions that finally prevail.

Summarizing our data, in the absence of an inflammatory stimulus, E_2_, at low concentrations resembling those in the serum of postmenopausal women receiving estrogen replacement therapy, exerts atheroprotective effects by mainly decreasing MMP-2 activity and increasing LOX expression via GPR-30 and by reducing MCP-1 protein expression via ERβ. The overexpression of ERα may result in E_2_-induced plaque instability by increasing the MCP-1 protein expression and MMP-2 activity. Significantly, in a low-grade inflammation state, such as established atherosclerosis, E_2_ promotes the destabilization of the atherosclerotic plaque by inducing the expression of molecules such as MCP-1 and MMP-9 and by increasing the activity of MMP-2 in the endothelial cells. These effects appear to be mediated via GPR-30. Moreover, p21 silencing results in equivocal effects on the expression of molecules involved in plaque vulnerability.

In conclusion, E_2_ induced different effects regarding atheromatous plaque instability through different ERs. The balance of the expression of the various ER subtypes may play an important role in the paradoxical characterization of estrogens as both beneficial and harmful.

To the best of our knowledge, this is the first study to assess, in vitro, the interrelation between different expression profiles of estrogen receptors and their regulatory effects on the expression of endothelial-derived factors implicated in atherosclerotic plaque vulnerability, addressing, at the same time, the possible involvement of p21 in this process. The strengths of this study are: (1) the performance of our experiments using aortic (arterial) endothelial cells (HAECs), which offer the best endothelial in vitro model system for studying the progression of atherosclerosis; (2) the fact that we incubated our cells with concentrations of E_2_ that resemble those observed in the serum of postmenopausal women receiving estrogen replacement therapy; and (3) the fact that we pre-incubated the aortic endothelial cells with TNF-α at concentrations that mimic the low-grade inflammation state observed in postmenopausal women with established atherosclerosis. The main limitation of our study is that we failed to assess (due to the HAECs’ sensitivity to the double transfection procedure) the role of p21 in the expression profiles of endothelial-derived factors involved in plaque vulnerability in the presence of either ERα or Erβ or both.

Further in vitro studies on other cellular components (i.e., VSMCs, immune cells) participating in the later stages of the atherosclerosis process, as well as studies on atherosclerosis animal models mimicking the postmenopausal status, are needed in order to delineate the exact roles of estrogen and of each estrogen receptor subtype in the atherosclerotic inflammatory process, so as to develop specific ER agonists/antagonists with an improved benefit/risk ratio.

## 4. Materials and Methods

### 4.1. Cell Culture and Treatment

Human aortic endothelial cells (HAECs) were purchased from Lonza and cultured in M200 medium (Gibco; Thermo Fisher Scientific, Inc., Waltham, MA, USA) supplemented with 10% fetal bovine serum (Gibco; Thermo Fisher Scientific, Inc.), 10% low-serum growth supplement (Gibco; Thermo Fisher Scientific, Inc.), and antibiotics (1% penicillin/streptomycin (Invitrogen; Thermo Fisher Scientific, Inc., Waltham, MA, USA). Cells were cultured in a cell incubator, providing a humidified environment with 5% CO_2_ and 95% air at 37 °C. Confluent four- to seven-passage HAECs were used in all the experiments. 

### 4.2. MTT Assay

Endothelial cells were plated 16 h before treatment on a 96-well plate at a cell density of 1 × 10^4^ cells per well. Cells were then incubated with various concentrations of E_2_ (10^−10^−10^−7^ M) (Cat. No: E2758, Sigma-Aldrich, St. Louis, MO, USA) and 2 ng/mL TNF-α alone for 24 h. The percentage of viable cells was measured using 0.5 mg/mL thiazolyl blue tetrazolium bromide (MTT). After 3 h of incubation at 37 °C, the MTT solution was removed and 100 µL of isopropanol was added to each well to aid the crystal dissolution. The optical density (OD) values of the colorimetric changes were measured using an ELISA reader at 570 nm.

### 4.3. Transfection with Small Interfering RNA

The HAECs were transfected at a 70% confluence for 24 h with 20 nM small interfering (si) RNAs targeting human p21 (sc-29427, Santa Cruz Technology, Dallas, TX, USA) using Lipofectamine 2000 reagent (Invitrogen) according to the manufacturer’s instructions. After 6 h, fresh medium was added, and the cells were incubated in full medium for further 24 h. Next, the cells were incubated with various concentrations of E_2_ (10^−10^−10^−7^ M) for 24 h prior to analysis.

### 4.4. Transient Transfection Assays

Next, 1 × 10^5^ HAEC were seeded in each well of the 12-well plate in 1 mL complete media. After 24 h, 1 μg/mL of the either ERα, ERβ, or a corresponding empty vector using DNA was introduced to the cells using AppliFect LowTox (A9027-applichem) transfection reagent according to the manufacturer’s instructions. Briefly, the cells were incubated with the DNA/AppliFect LowTox mix for 4 h, and then the media was replaced with fresh media for a further 20 h. Twenty-four after the transfection, the media was switched to 5% charcoal stripped FBS (Gibco) phenol-free complete media for another 6 h, followed by the incubation of the cells with the various concentrations of E_2_ (10^−10^−10^−7^ M) for a further 24 h. The efficiency of the transfection was determined by qPCR and western blot analysis. All experiments were repeated a minimum of three times.

### 4.5. RNA Isolation and qPCR

The qRT-PCR was performed as previously described [121]. For the quantitative real-time PCR, HAEC cells were plated on 12-well plates 16 h prior to treatment with various concentrations of E_2_ (10^−10^−10^−7^ M) for periods of 6, 12, and 24 h. The cells were also pre-incubated with TNF-α (2 ng/mL) for 24 h in order to mimic the low-grade inflammation seen in atherosclerosis, and then co-incubated with various concentrations of E_2_. At the end of the treatment, the cells were harvested and the total RNA was isolated from the HAECs using NucleoSpin^®^ RNA Plus (Macherey-Nagel, Düren, Germany). The quality of the extracted mRNA was evaluated by nanodrop. A volume equal to 1000 ng of RNA was then reverse-transcribed using the LunaScript™ RT SuperMix Kit (New England Biolabs, Ipswich, MA, USA) in accordance with the manufacturer’s instructions. Glyceraldehyde 3-phosphate dehydrogenase (GAPDH) was used as a normalization control. The mRNA levels of GAPDH, MMP2, MMP-9, TIMP-1, TIMP-2, MCP-1, OPG, RANK, RANKL, ADAMTS-4, lysyl oxidase (LOX), PDGF-,β and p21, as well as the presence of estrogen receptors ERα, Erβ, and GPR-30 in the HAEC cells, were evaluated using the SYBR Green-based quantitative real-time polymerase chain reaction (qRT-PCR) protocol on a CFX96 (Biorad). The 2−ΔΔCT method was used to determine the expression level. The sequence of the primers used in this study is listed in Table 1. All experiments were performed in triplicate.

### 4.6. SDS-PAGE and Western-Blot Analysis

The western blot analysis was performed as previously described [121]. Briefly, whole-cell lysates were prepared in lysis buffer (Cell-Signaling Technology, Boston, MA, USA). Samples containing 30 μg of protein were resolved using electrophoresis gels and transferred to a nitrocellulose membrane. The membranes were blocked for 1 h with 5% skimmed milk in PBS with 0.1% Tween 20 and subsequently incubated overnight at 4 °C with anti-ERα (sc-8002, Santa Cruz Technology, Dallas, TX, USA, 1:800), anti-ERβ (sc-390243, Santa Cruz Technology, Dallas, TX, USA, 1:800) anti-GPR30 (sc-48524-R, Santa Cruz Technology, 1:800), anti-p21 (Cell-Signaling, MA, USA, 1:500), and anti-β-actin (Millipore Corporation, Billerica, MA, USA, 1:5000) primary antibodies. The membranes were probed with secondary antibodies coupled with horseradish peroxidase goat anti-mouse IgG-HRP (31430, Thermo Scientific; 1:2500) and goat anti-rabbit IgG-HRP (AP132P, Millipore Corporation, Billerica, MA, USA, 1:2500) at room temperature (RT) for 1 h. The detection of the immunoreactive bands was performed using Clarity Western ECL Substrate (BioRad, Bio-Rad Laboratories Inc., Hercules, CA, USA). For this purpose, β-actin served as a loading control. The densitometric analysis was performed using Image J.

### 4.7. ELISA

Cell-secreted TIMP-1 (DTM100, R&D Systems, Minneapolis, MN, USA), TIMP-2 (DTM200, R&D Systems, Minneapolis, MN, USA), MCP-1 (DCP00, R&D Systems, Minneapolis, MN, USA), and OPG (RAB0484, Sigma-Aldrich, St. Louis, Missouri, USA) were measured with the respective ELISA kits containing pre-coated ELISA plates, and the assays were performed as described by the manufacturers. Briefly, 100 μL of cell culture supernatant or standard was incubated in each well for 3 h at RT. Then, the HRP-conjugated antibody was added and incubated for 1 h at RT, followed by aspiration and three washes. Next, horseradish peroxidase was added and incubated for 1 h, followed by aspiration and washes. 3,3′,5,5′-Tetramethylbenzidine substrate was added to each well and incubated for 30 min in a dark chamber. Stop solution was added, and the absorbency of all ELISAs was read at 450 nm with a plate reader (Bio-Rad^®^ Microplate Absorbance Reader, Bio-Rad Laboratories Inc., Hercules, CA, USA).

### 4.8. Zymography

The gelatin zymography activity of MMP-2 was evaluated by measuring the gelatinolytic activities of pro-MMP-2 and active MMP-2. Equal numbers of HAECs (1 × 10^−6^ cells/well) were cultured in M200 medium for 24 h, and then either normal HAECs or cells transfected with ERα, ΕRβ, plasmid, or p21 siRNA were serum-starved for 16 h. Thereafter, the cells were incubated with various concentrations of E_2_ (10^−10^−10^−7^ M) alone (24 h) or pre-incubated with TNF-α (2 ng/mL) for 24 h and then co-incubated with various concentrations of E_2_ for a further 24 h. At the end of the incubation time, the cell supernatant was collected and concentrated using Amicon Ultra centrifugal filters (30 kDa-Millipore). The protein concentrations were calculated by performing a Bradford assay. Pro-MMP-2 and active MMP-2 proteins in the conditioned media were separated without prior boiling by electrophoresis with 10% sodium dodecyl sulfatepolyacrylamide gels containing 0.1% (weight/volume) gelatin (Sigma-Aldrich). The gels were incubated with 2.5% Triton X-100 for 1 h at room temperature. The gels were then incubated at 37 °C in the developing buffer (Invitrogen) for 16 h. The gels were stained with 0.5% Coomassie Brilliant Blue (AppliChem, Darmstadt, Germany) and de-stained in a solution containing 40% methanol and 10% acetic acid. Clear zones against the blue background indicated the presence of gelatinolytic activity. The densitometrical analyses of the zymographic images were performed using image J software (NIH, Bethesda, MD, USA).

### 4.9. Statistical

Data are represented as mean ± SD. The statistical analysis was performed using Student’s *t*-test with a two-tailed distribution. The statistical analysis of the real-time PCR data was performed using the non-parametric test (Wilcoxon signed rank test). All statistical analyses were performed using GraphPad Prism 7 Software. In all conditions, the minimum level of significance was set at *p* < 0.05.

## Figures and Tables

**Table 1:** The sequences of primers used for the qRT-PCR analysis.

Primer	Forward	Reverse
**MMP-2**	5′-TGGCAAGTACGGCTTCTGTC-3′	5′-TTCTTGTCGCGGTCGTAGTC-3′
**MMP-9**	5′-TGCGCTACCACCTCGAACTT-3′	5′-GATGCCATTGACGTCGTCCT-3′
**TIMP-1**	5′-TGCGGATACTTCCACAGGTC-3′	5′-GCATTCCTCACAGCCAACAG-3′
**TIMP-2**	5′-AAGAGCCTGAACCACAGGTA-3′	5′-GAGCCGTCACTTCTCTTGAT-3′
**MCP-1**	5′-AATAGGAAGATCTCAGTGCA-3′	5′-TCAAGTCTTCGGAGTTTGGG-3′
**OPG**	5′-GGAACCCCAGAGCGAAATACA-3′	5′-CCTGAAGAATGCCTCCTCACA-3′
**RANK**	5′-CCCGTTGCAGCTCAACAAG-3′	5′-GCATTTGTCCGTGGAGGAA-3′
**RANKL**	5′ -ACGCAGTGAAAACACAGTT-3′	5′-TGCCTCTGGCTGGAAACC-3′
**LOX-1**	5′ -CCAGAGGAGAGTGGCTGAAG-3′	5′-CCAGGTAGCTGGGGTTTACA-3′
**PDGF- β**	5′-CCATTCCCGAGGAGCTTTATG-3′	5′-CAGCAGGCGTTGGAGATCAT-3′
**P21**	5′-ATGAAATTCACCCCCTTTCC-3′	5′-CCCTAGGCTGTGCTCACTTC-3′
**ER-α**	5′-TGGGCTTACTGACCAACCTG-3′	5′-CCTGATCATGGAGGGTCAAA-3′
**ΕR-β**	5′-AGAGTCCCTGGTGTGAAGCA-3′	5′-GACAGCGCAGAAGTGAGCATC-3′
**GPR-30**	5′-TCACGGGCCACATTGTCAACCTC	5′-GCTGAACCTCACATCTGACTGCTC
**GAPDH**	5′-GGGTGTGAACCATGAGAAGT-3′	5′-CATGCCAGTGAGCTTCCCGTT-3′
**ADAMTS-4**	5′-GACACTGGTGGTGGCAGATG-3′	5′-TCACTGTTAGCAGGTAGCGCTTTA-3′

## Data Availability

Data are contained within the article and its Supplementary Materials.

## Supplementary Materials

The following supporting information can be downloaded at: https://www.mdpi.com/article/10.3390/ijms231810960/s1.
