# Supplementary material for: Estrogen Receptor Subtypes Elicit a Distinct Gene Expression Profile of Endothelial-Derived Factors Implicated in Atherosclerotic Plaque Vulnerability
Source: Int J Mol Sci. 2022 Sep 19;23(18):10960. doi: 10.3390/ijms231810960 (PMC9506323; doi:10.3390/ijms231810960)
Supplement: Supplementary file 1 [file ijms-23-10960-s001.zip › ijms-1888928 - supplementary.pdf]

International Journal of
Molecular Sciences

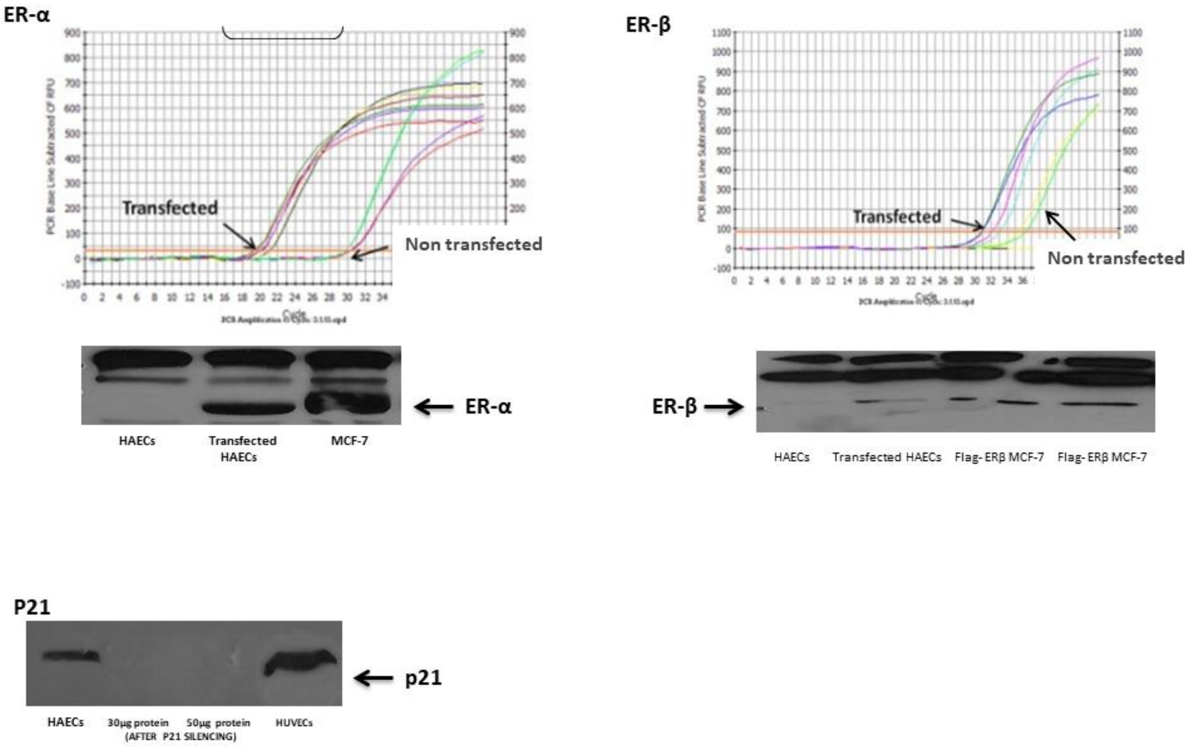

Figure S1. The efficiency of ER-α and ER-β transfection as well as P21 silencing was evaluated by qPCR and Western blot analysis.
